# Submaxillary Mucin: its Effect on Aroma Release from Acidic Drinks and New Insight into the Effect of Aroma Compounds on its Macromolecular Integrity

**DOI:** 10.1007/s11483-019-09574-2

**Published:** 2019-04-15

**Authors:** Vlad Dinu, Richard B. Gillis, Thomas MacCalman, Mui Lim, Gary G. Adams, Stephen E. Harding, Ian D. Fisk

**Affiliations:** 1 National Centre for Macromolecular Hydrodynamics, University of Nottingham, Sutton Bonington Campus, Leicestershire, UK; 2 Division of Food Sciences, School of Biosciences, University of Nottingham, Sutton Bonington Campus, Sutton Bonington, Leicestershire, UK; 3 School of Health Sciences, Faculty of Medicine and Health Sciences, Queen’s Medical Centre, Clifton Boulevard, Nottingham, UK; 4 Kulturhistorisk Museum, Universitetet i Oslo, Postboks 6762, St. Olavs plass, 0130 Oslo, Norway

**Keywords:** Bovine submaxillary mucin, Aroma release, AUC, SEC-MALS, Acidic drinks

## Abstract

Submaxillary mucin is a major component that defines the makeup and functionality of saliva. Understanding its structure and function during food intake is key to designing appropriate strategies for enhancing the delivery of flavour. In the present study, the hydrodynamic integrity of bovine submaxillary mucin was characterised under physiological and acidic conditions and it was shown to have a broad molecular weight distribution with species ranging from 100 kDa to over 2000 kDa, and a random coil type of conformation. A decrease in the pH of mucin appeared to result in aggregation and a broader molecular weight distribution, which was shown to correlate with a release of flavour compounds. Our study also provides indications that *p*-cresol may have an effect on the macromolecular integrity of mucin.

## Introduction

Past research has generally focused on the behaviour of volatile aroma compounds in the presence of small solutes i.e. monosaccharides, salts and proteins [1, 2]. However, there is limited research on the effect of saliva and its macromolecular constituents on aroma release. During consumption of food, saliva is the first medium encountered by aroma compounds as they travel to the respiratory tract and to the olfactory receptors in the nose [3]. Saliva consists of a number of macromolecular components which protect and lubricate the lining of the oral cavity: mucins, amylases, proteins and antibodies, which play a role in regulating pH and normal functioning of the salivary film [[4, 5]. Mucin glycoproteins are the second most abundant salivary components, after salivary α-amylase, and define the structure and functionality of saliva [6]. Although the pH of the saliva is maintained between ~5.5 to 7.5, this can temporarily decrease during consumption of foods, interfering with the normal functioning of mucins and other salivary proteins [7]. These salivary changes trigger salivary glands to produce more saliva in order to compensate for the loss in functionality and to keep the oral cavity lubricated [8]. But the process is faster at replacing low molecular weight enzymes and peptides, and rather slow at replenishing viscous, higher molecular weight mucins, therefore stimulated saliva has lower mucin levels than resting saliva [5]. This effect is predicted to influence the partitioning of aroma compounds from the saliva to the olfactory receptors.

Aroma release is affected by a number of physio-chemical interactions. These effects can lower the concentrations of aroma compounds released from the bolus, resulting in a significant loss through ingestion [9, 10]. Therefore, to enhance flavour release in vivo, it is required to understand the behaviour of salivary components under different conditions i.e. pH, salt, temperature. Ultimately, these conditions are influenced by the physiochemical properties of the ingested product, such as the pH of soft drinks and other foods, which can be as low as 2.4. Recent studies on mucins have suggested that acidic environments can induce conformational changes [11]. This because mucins have a pH dependant negative charge, progressively falling as the pH decreases [12]. These pH-mucin changes are proposed to promote the release of volatile aroma compounds. In addition, some studies showed that sugars promote the degree of acid production via citrates/citric acid present in drinks interacting with the calcium present in the tooth, thus maintaining a lower pH over longer periods of time [13, 14]. Other studies have shown that the partitioning of aroma compounds is lower in the absence of sucrose [2], and this suggests that the lack of sucrose is also responsible for the lack of flavour release in ‘low calorie’ drinks, despite their reformulation.

Mucins from the alimentary, reproductive and bronchial tracts are characterised as having large molecular weights of up to 20 million Daltons (Da, or in molar mass terms, g/mol), although values up to 50 million Da have also been reported [15, 16]. They adopt a branched, random coil type of conformation as described by various techniques such as AUC (analytical ultracentrifugation), SEC-MALS (size-exclusion chromatography coupled to multi-angle laser light scattering), AFM (atomic force microscopy) or electron microscopy [16, 17]. They resemble block copolymer structures, which contain a polypeptide backbone, abundant in serine, proline and threonine, which forms a bridge between the hydroxyl groups of the carbohydrate fraction. The carbohydrates can account for up to 90% of the entire molecular weight of mucin and is generally made up of galactose, sialic acid, fucose, N-acetyl galactosamine and N-acetyl glucosamine. These highly hydrophilic coated regions alternate with hydrophobic and non-glycosylated protein domains [18]. Protein domains, which are responsible for mucin polymerisation, are susceptible to proteolytic cleavage [19]. Recent studies looked at the formation of the muco-salivary film and suggested that it is formed by a monomer/dimer type of interaction in a lateral or ‘layer-by-layer’ approach, such that subunits are bridged together via their cysteine-rich protein domains [20].

Salivary/submaxillary mucins are different from alimentary, reproductive and tracheobronchial mucins. They have lower molecular weights, lower carbohydrate content and up to 30% more charged sialic acid residues [21]. Because human salivary/submaxillary mucin is difficult to obtain in any useful quantities, bovine submaxillary mucin (BSM) was used as a surrogate in the current study. Experiments on submaxillary mucin were amongst the first to be carried to investigate mucin’s complex structural assembly [22, 23]. It appears that the glycosylated region makes up to 60% of the total mass, with abundance in acidic disaccharides such as sialic acids and N-acetyl galactosamines [24]. A detailed analysis of its oligosaccharide region has been reviewed by Chai, et al. [25].

The current study aims to investigate the molecular interactions between volatile aroma compounds and submaxillary mucin under neutral and acidic conditions. Mucin solutions were designed to mimic the physiological conditions of the saliva (buffered salts and electrolytes) and within the range of salivary concentration of mucin (0–2 mg/ml) [26, 27]. Analytical Ultracentrifugation (AUC), SEC-MALS (size-exclusion chromatography coupled to multi-angle laser light scattering), viscometry, Dynamic Light Scattering (DLS) and Raman spectroscopy were used to explore changes in mucin integrity. The hydrodynamic analysis has been linked with Atmospheric Pressure-Chemical Ionization Mass Spectrometry (APCI-MS) to explain changes in the aroma release. This strategy was further complemented by Raman spectroscopy to gain a better understanding of the interactions of an aroma active compounds- *p*-cresol with mucin.

## Materials and Methods

### Materials

Bovine submaxillary mucin (Type IS) and volatile aroma compounds were purchased from Sigma Aldrich (Dorset, UK). Highly purified RO (reverse osmosis) water was used for the preparation of buffer solutions. The 0.1 M phosphate buffered saline (PBS) was made according to Green (1933) [28] (Fisher Scientific, UK). The Na-citrate buffer solutions (pH 2.6–5) were made by varying the proportion of citric acid and sodium phosphate. Solutions of mucin have been dialysed with a 14,000 Da membrane (Fisher Scientific) and filtered through 0.45 μm Whatman nylon membrane filters (Millipore).

### Methods

#### Sedimentation Velocity in the Analytical Ultracentrifugation (AUC)

Experiments were performed at 20.0 °C using the Optima XL-I analytical ultracentrifuge (Beckman, Palo Alto, USA) equipped with Rayleigh interference optics. Samples of 395 μl (and 405 μl solvent) were injected into 12 mm double sector epoxy cells with sapphire windows and centrifuged at 30000 rpm. The interference system produced data derived by recording changes in concentration (in fringe units) versus radial displacement. Data were analysed in SEDFIT using the “least squares *g*(*s*)’ method of Dam & Schuck [29] by generating sedimentation coefficient distributions, g(*s*) vs *s*, where *s* is the sedimentation coefficient (in Svedberg units, S = 10 ^−13^ s). Weight average sedimentation coefficient values from the *g*(*s*) vs *s* distributions were normalised to standard conditions (viscosity and density of water at 20.0 °C) to give s_*20,w*_ [30]. A partial specific volume of 0.66 ml/g was employed. To eliminate non-ideality, sedimentation coefficients are extrapolated to zero concentration, c using standard 1/*s*_*20,w*_ vs *c* plots.

#### SEC-MALS-Viscostar (Size-Exclusion Chromatography Coupled to Multi-Angle Laser Light Scattering and Viscometer)

SEC-MALS has been a powerful method for the evaluation of molecular weight distribution of mucins since the 1st application by Jumel, Fiebrig & Harding in 1996) [31], and was used for evaluation of mucin integrity at pH 7.0. The SEC set-up consisted of a Postnova Analysis PN7505 degassing unit (Landsberg am Lech Germany), Shimadzu LC-10 AD HPLC Pump (Shimadzu UK, Milton Keynes, UK.), fitted with a Spark-Holland Marathon Basic autosampler (Spark Holland, Emmen, The Netherlands) combined with a TSK Gel guard column (7.5 × 75 mm) and TSK Gel G5000, G6000 columns (7.5 × 300 mm) connected in series (Tosoh Biosciences, Tokyo, Japan), fully flushed of column debris. Light scattering intensities were simultaneously detected at 18 angles as a function of elution volume using a DAWN® HELEOS™ II, light scattering photometer connected in series to a ViscoStar® II on-line differential viscometer, an Optilab® rEX refractive index detector (Wyatt Technology Corporation, California, U.S.A.). A stock solution of 1.0 mg/ml was filtered through a 0.45 μm syringe filter (Whatman, Maidstone, England) - to remove any insoluble material or dust prior to injection - and then injected into the autosampler. A 100 μL aliquot of each solution was injected onto the columns at ambient temperature. The eluent employed was the PBS dialysate at a flow rate of 0.8 ml/min. ASTRA™ (Version 6) software (Wyatt Technology Corporation, Santa Barbara, U.S.A.) was used to estimate the weight average molecular weight, *M*_w_, weight average intrinsic viscosity [η] and radius of gyration *R*_g_, and also molecular weight, *M*(*V*_e_), intrinsic viscosity [η](*V*_e_) and the radius of gyration *R*_g_(*V*_e_) as a function of elution volume *V*_e_. A 4 mW He-Ne laser was used at a wavelength of 632.8 nm, and the refractive increment for the mucin was taken as 0.181 ml/g. Because of the low solute concentrations after dilution on the columns non-ideality effects were assumed as negligible. Due to column limitations evaluations were not possible at pH 3, instead standard capillary viscometry was employed at a temperature of 20.0 °C [32].

#### Extended Fujita Method for Molecular Weight Distribution

Comparative molecular weight distributions *f*(*M*) vs *M* for BSM in solvents at pH 7.0 and pH 3.0 were obtained using the Extended Fujita method of Harding, Schuck and colleagues [33], which converts g(*s*) vs s plots to *f*(*M*) vs *M* and uses the scaling relationship

1 $$ s={\upkappa}_{\mathrm{s}}{M}^{\mathrm{b}} $$

where b depends on the conformation. To transform g(*s*) vs. *s* to f(*M*):

2 $$ f(M)=\mathrm{g}(s)\left( ds/ dM\right) $$

where

3 $$ ds/ dM=b.{\upkappa_{\mathrm{s}}}^{1/b}.{s}^{\left(b-1\right)/b} $$

Therefore, to perform the transformation the conformation type or *b* needs to be known under the particular solvent conditions and at least one pair of *s-M* values is needed to define the κ_s_ from Eq. (3). The method has been built into the SEDFIT algorithm along with the *g*(s) vs *s* procedure. To minimize non-ideality a very low loading concentration (*c* = 0.25 mg/ml) was chosen for the transformation.

#### Dynamic Light Scattering (DLS)

The experiments were performed using the Zetasizer Nano-ZS detector and low volume (ZEN0112) disposable sizing cuvettes (Malvern Instruments Ltd., Malvern, UK). The samples were measured at (20.0 ± 0.1) °C using the 173° scattering angle collected for 3 runs of 10 s at each mucin concentration (*c* = 0.2–1.0 mg/ml). Apparent z-average hydrodynamic radii, *r*_h,app_ were evaluated from the apparent translational diffusion coefficient, *D*_z,app_ via the Stokes-Einstein equation:

4 $$ {r}_{\mathrm{h},\mathrm{app}}=\left({\mathrm{k}}_{\mathrm{b}}T\right)/6\uppi \upeta {D}_{\mathrm{z},\mathrm{app}} $$

where k_*B*_ is the Boltzmann constant, η is the solution viscosity, T is the absolute temperature (K). We would like to stress that *D*_z,app_ (and *r*_h,app_) values are only apparent values as they were recorded at a scattering angle of 173^o^ and no angular extrapolation to zero angle was possible with the instrumentation to correct for rotational/ anisotropic diffusion effects [34].

#### Raman Spectroscopy

Raman was performed using a RamanRXN2 optical system (Kaiser, Boston, USA) and the following conditions: laser wavelength of 785 nm, laser power at sample of ~40 mW with a spectral resolution of 7.5 cm^−1^, fitted with a NCO-3.0-NIR system. The exposure was 30 s for the 10 mg/ml mucin sample. Raman spectra was not corrected for noise and the distribution is a result of an average of 10 scans, without smoothing. Due to the dilute state of the mucin preparation and the high proportion of carbohydrate fractions in mucin, it was difficult to obtain a higher resolution configuration [35].

#### Atmospheric Pressure Chemical Ionization-Mass Spectrometry (APCI-MS)

The APCI-MS (Platform II, Micromass, Manchester) was used to analyse the concentrations of volatile compounds above the headspace of the solutions under static conditions. A final concentration of ~10–50 ppm (parts per million) was sampled with an air flow adjusted to 10 ml/min. The instrument was set in Selective Ion Recording (SIR) mode to monitor the selected mass to charge ions (m/z) of the aroma compounds: 108.1, 128.13, 128.21, 156.2, 142.23 and 154.25. The ion intensity was measured at cone voltage of 50 V, source temperature of 75 °C and dwell time of 0.02 s. Sampling took place for 10 s, enough for the signal to plateau. Each peak was integrated in Mass Lynx (Waters, UK) and used to compare the maximum ion count for all samples using a similar approach used previously [36, 37].

#### Statistical Analysis

The AUC and all GC-MS samples were performed in triplicate and the analysis was prepared using Tukey’s post hoc test to identify significance (*P* < 0.05). All figures were prepared in Origin 7.5 (OriginLab, Massachusetts, USA).

## Results and Discussion

### Characterisation of Bovine Submaxillary Mucin (BSM)

**Fig. 1:**
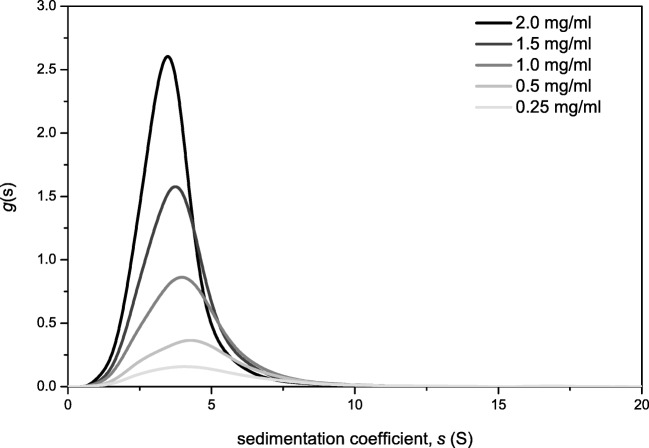
Sedimentation velocity, *g*(s) analysis of BSM showing the sedimentation coefficient distribution and the extrapolation to zero concentration. Run at 20.0 °C at 30000 rpm in PBS buffer 0.1 M (pH 7)

The sedimentation coefficient distribution of mucin was analysed by the *g*(s) method incorporated in SEDFIT [29, 38]. The analysis shown in Fig. 1 was used to estimate the weight average sedimentation coefficients which were corrected to standard conditions (density and viscosity of water at 20.0 °C) and then the reciprocals extrapolated to zero concentration s^o^_20*,w*_ (S) [38]. The experiment confirmed the presence of a polydisperse sample, similar to other mucins [39]. The s^o^_20*,w*_ values were commensurate with those found earlier by Payza and coworkers [39].

**Fig. 2:**
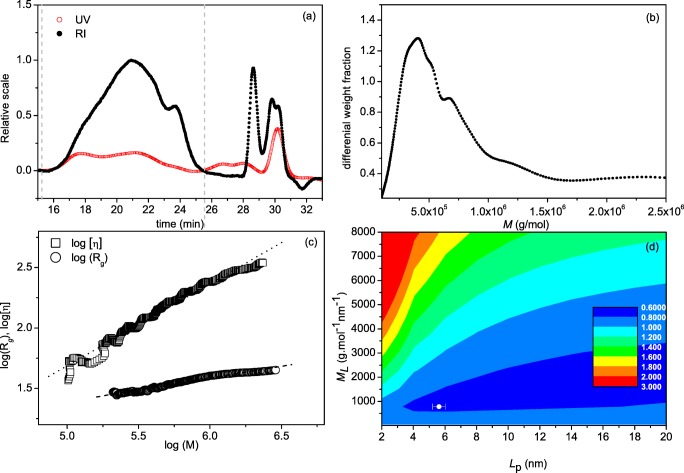
SEC-MALS analysis of bovine submaxillary mucin. **a** Concentration (refractive index) and ultra-violet (UV) elution profiles; **b** molecular weight distribution; **c** Mark Houwink-Kuhn Sakurada (MHKS) plot of intrinsic viscosity [η](*V*_e_) vs molecular weight *M*_w_(*V*_e_) at different elution volumes, *V*_e_. [η](*V*_e_), versus molecular weight *M*_w_(*V*_e_) and radius of gyration *R*_g_(*V*_e_) vs *M*_w_(*V*_e_). **d** Multi-HYDFIT contour plot to obtain estimates for the flexibility parameter the persistence length *L*_p_ and the mass per unit length *M*_L_

**Fig. 3:**
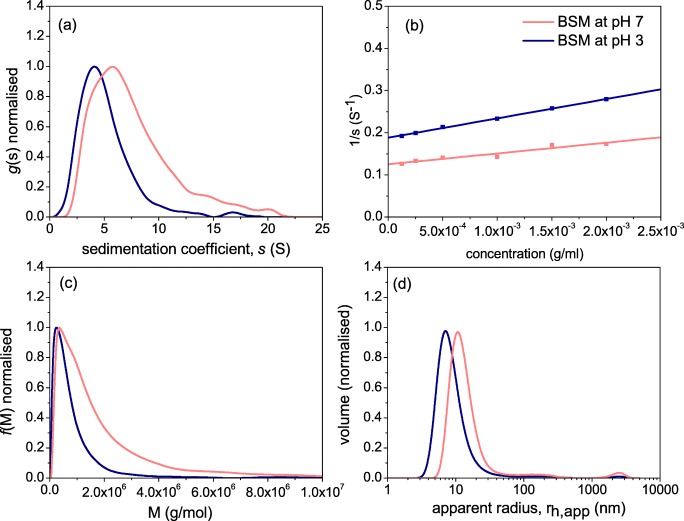
Comparative hydrodynamic properties of bovine submaxillary mucin at pH 7.0 and at pH 3.0 (**a**) sedimentation coefficient *g*(s) distribution at 0.25 mg/ml; **b** reciprocal plot of s versus concentration, fitted to (1/*s*) = (1/*s*°)(1 + *k*_s_*c*) where *k*_s_ is the concentration dependence ‘Gralén’ coefficient [47]; **c** molecular weight distribution from sedimentation velocity and Extended Fujita analysis, *f*(*M*) versus M for a loading concentration of 0.25 mg/ml. κ_s_ = 0.007606 and *b* = 0.483; **d** Distribution of the apparent hydrodynamic radius *r*_h,app_. Experiments performed in PBS buffer 0.1 M (pH 7.0) and Na-citrate buffer 0.1 M (pH 3.0) at a temperature of 20.0 °C

SEC-MALS was then applied to BSM (Fig. 2a) revealing the Rayleigh Interference (RI) and UV elution profiles of BSM in 0.1 M PBS (pH 7.0), ranging from elution times of 16 min to 25 min. The elution profiles are converted to a molecular weight distribution profile in ATRA™ software, as supplied by the manufacturer (Fig. 2b) which yielded a M_w_ value of ~700 kDa. The broad distribution was consistent with the multi-component nature of the sedimentation coefficient analysis. Data from SEC-MALS was also analysed for the comparison of molar mass and intrinsic viscosity, [η]. The double logarithmic plot between the two variables can be used to estimate the conformation of the macromolecule (Fig. 2d). The samples show good correlation between molar mass and intrinsic viscosity, consistent with those found for other mucins [40, 41]. A more quantitative representation comes from the measurement of the persistence length *L*_p_, which has theoretical limits of 0 for a random coils and ∞ for a stiff rod, and mass per unit length, *M*_L_ [42]. In this study, *L*_p_ and *M*_L_ have been estimated using the MultiHYDFIT package, incorporating the Yamakawa-Fujii [43] and the Bushin–Bohdanecky [44, 45] relations into a single ‘global’ algorithm that can be used to estimate *L*_p_ and *M*_L_ based on a minimisation of a target function (Δ), which considers parameters including sedimentation coefficients, radius of gyration *R*_g_, and intrinsic viscosity [η], for the entire molecular weight distribution [40, 46]. Our results yielded a persistence length value, *L*_p_ of (5.6 ± 0.5) nm and mass per unit length, *M*_L_ of ~800 g.mol^−1^.nm^−1^, corresponding to a random coil conformation for the BSM mucin molecules (Fig. 3d), as found for other mucins [39].

### Effect of pH on the Hydrodynamic Properties of BSM

**Fig. 4:**
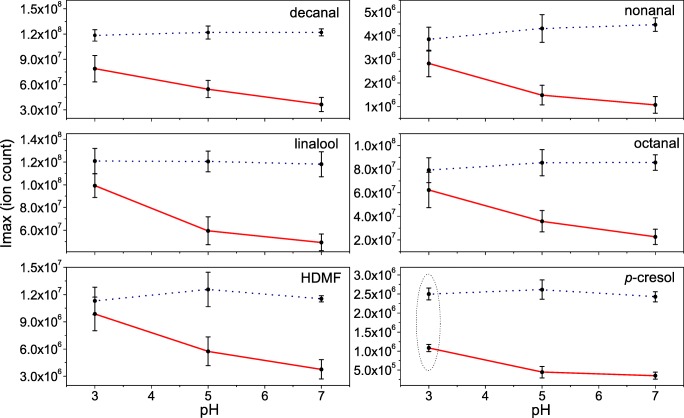
APCI-MS results showing the impact of pH on the release of volatile aroma compounds from solutions with mucin (1 mg/ml) and from the neutral and acidic buffer solutions (dotted line). The data shown are given as mean + standard error, number of observations, *n* = 3

The AUC and DLS results of Fig. 3 demonstrate how lowering the pH can affect the hydrodynamic properties of mucin. Sedimentation velocity *g*(s) analysis (Fig. 4a) revealed that the macromolecular distribution of mucin has shifted towards higher *s*°_20,w_ values, from 5.2S at pH 7.0 to 7.9S at pH 3. The reciprocal sedimentation coefficients were extrapolated to zero concentration to determi.0e sedimentation concentration dependence coefficient k_s_, which were (238 ± 9) ml/g at pH 7.0 and (202 ± 18) ml/g at pH 3.0 (Fig. 4b and Table 1). The “Extended Fujita” *f*(*M*) vs *M* transformation from g(*s*) vs *s* was performed as according to Harding et al. [48, 49]. The procedure is employed by calculating the κ_s_ and *b* parameters, the latter being determined from the plot of molar mass and intrinsic viscosity, [η], first yielding the viscosity power law (scaling) coefficient *a*, which corresponds to a value of (0.565 + 0.010), equivalent to a random coiled conformation. We then use the Tsvetkov et al relation [50] linking the sedimentation and viscosity power law coefficients

5 $$ b=\left(2-a\right)/3 $$

to obtain a value of *b* = (0.483 ± 0.010). In order to determine κ_s_, we used the weight average sedimentation coefficient *s*^o^_20,w_, together with the weight average *M*_w_ from SEC-MALS, and the *b* value, as follows:

6 $$ {\upkappa}_{\mathrm{s}}=s/{M}^b $$

and obtained a κ_s_ value of 0.007606. Encouragingly the molecular weight distribution from the Extended Fujita analysis in Fig. 3c is similar to that from SEC MALS (Fig. 2b) at pH 7.0. This gives us confidence that the shift to higher molecular weights at lower pH is genuine.

**Table 1:** Hydrodynamic properties of bovine submaxillary mucin in 0.1 M phosphate chloride buffer (pH 7.0) and citrate buffer (pH 3.0)

	pH 7.0	pH 3.0
*s*^o^_20,w_ (S)	5.2 (±0.2)	7.9 (±0.7)
*M*_w,app_ (kDa)	700 (±30)	1600 (±160)
*r*_h,app_ (nm)	10 (±3)	15 (±3)
*k*_s_ (ml/g)	238 (±9)	202 (±18)
[η] (ml/g)	168 (±5)	127 (±2)
*R* = *k*_s_/[η]	1.4 (±0.2)	1.6 (±0.3)
*R*_g_ (nm)	35.1(±1.2)	–
*L*_p_ (nm)	5.6 (+0.5)	–

Figure 3d shows the apparent hydrodynamic radius of BSM by volume, uncorrected for concentration and rotational diffusion effects [34]. The analysis produced uniform size distributions across all concentrations with an apparent hydrodynamic radius, *r*_h_,_app_ of ~10 nm at neutral pH, corresponding to an apparent diffusion coefficient *D*_app_ of 2.2 × 10^−7^ cm^2^/s. Conversely, the low pH fractions also reveal a shift to higher apparent hydrodynamic sizes, supporting the results from sedimentation velocity (Fig. 3d). We stress that the these values are apparent values as they are given only for comparison and are not genuine radii since there has been no angular dependence correction due to rotational diffusion effects [34].

Generally, the SEC-MALS and AUC analyses (i) confirm the low weight average molecular weight ***M***_w_, of BSM at pH 7.0 is ~700 kDa, as compared to other mucins from other sources [51, 52], but with the presence of a high molecular weight tail extending to several million Da. (ii) the shift to high molecular weights (and higher apparent hydrodynamic radii) at pH 3.0 is clear from Fig. 3c and Table 1, where the distribution weight average *M*_w_, has increased to ~1600 kDa. This behaviour is not unexpected as the lowering of pH to near the pKa of sialic acid means reduced overall negative charge and hence greater tendency to aggregate. This is somewhat similar to the behaviour observed in gastric mucus gelation, used to protect the stomach from auto-digestion, although one major difference is that salivary/submaxillary mucins have a much higher proportion of protein [53].

We can also address the question as to whether this increase in molecular weight is accompanied by a change in conformation. The simplest indicator of conformation comes from the Wales and van Holde [54] ratio ‘*R*’, in which the ratio of the sedimentation concentration dependence coefficient *k*_s_, to the intrinsic viscosity [η], is a direct measure (independent of assumptions of hydration) of the macromolecular gross conformation in solution. The *R* values reported in this study 1.4 ± 0.2 and 1.6 ± 0.3 at pH 7.0 and pH 3.0 respectively both corresponding to random coils, although within experimental error they are indistinguishable at the 2 pH’s, the results indicate a change to a more compact conformation [55, 56]. The value at pH 7 is also consistent with a MHKS *a* coefficient value of (0.565 + 0.010), and persistence length *L*_p_ = (5.6 + 0.5) nm, obtained from HYDFIT analysis. The SEC-MALS-viscostar and sedimentation velocity results are all consistent with a random coil. It is worth suggesting that these mild changes in the macrostructure of mucus at low pH may also correspond the mild feeling of astringency perceived during consumption of soft drinks.

### Interactions of Volatile Aroma Compounds with Mucin Solutions

Figure 4 shows the effect of pH on the partitioning of six characteristic volatile compounds found in lemon flavoured soft drinks in the presence and absence of mucin. In the absence of mucin, results indicated no significant differences (*P* > 0.05) in the release of these compounds at different pH (Fig. 4-dotted line), thus excluding the possibility of significant solvent effect. However, there was a substantial interaction effect which lowered the partitioning of aroma volatiles in the presence of mucin. For most compounds, this effect was pH dependent, with no significant differences at pH 3.0, but with significant differences (*P* < 0.05) observed for pH 5 and pH 7, indicating that pH-mucin effects lower the damping effect of mucin on the release of aroma. Interestingly, *p*-cresol showed a ~2.5 fold decrease in the relative concentration on the addition of mucin, irrespective of pH. It is known that some proteins can bind specific aroma compounds [13, 57, 58], hence the significant reduction in the release *p*-cresol indicates a possible interaction mechanism with mucin.

**Fig. 5:**
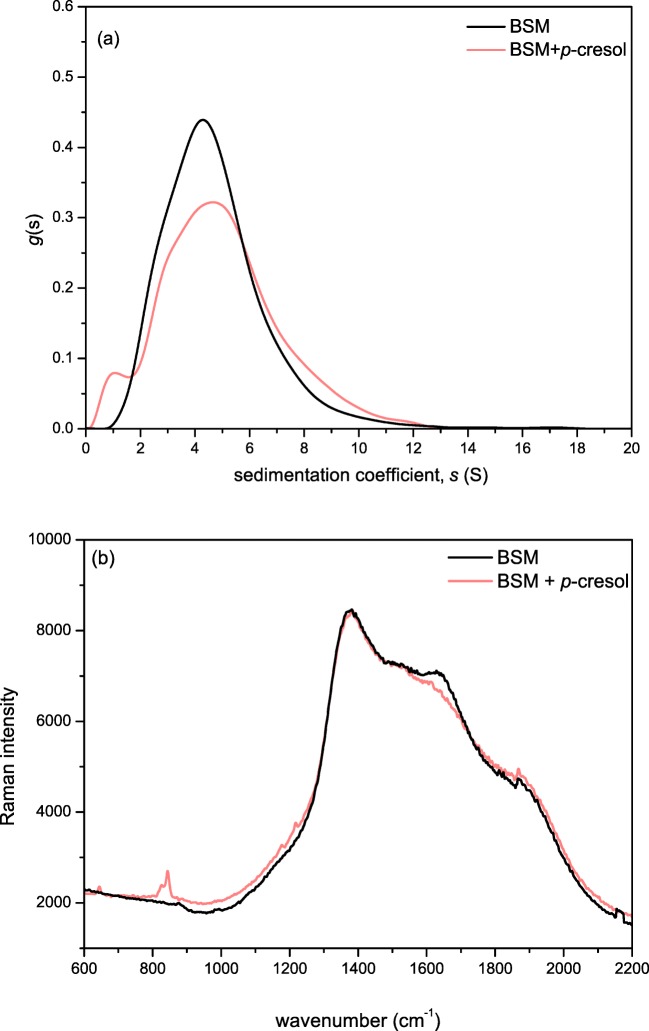
**a** Sedimentation coefficient distribution *g*(s) vs s profiles for bovine submaxillary albumin (loading concentration *c* = 0.25 mg/ml) and the result of its interaction with *p*-cresol (*c* = 0.1 mg/ml); **b** Raman spectra of (*c* = 10 mg/ml) with and without *p*-cresol. The mucin/*p*-cresol sample was dialysed against a 14 kDa membrane prior to Raman analysis to remove unbound *p*-cresol. Performed in 0.1 M PBS pH 7 at 20.0 °C

Para-cresol (*p*-cresol) and its interactions with mucin has not been previously documented. This is of particular interest as it one of the main products resulting from the degradation of citral, and can be found in a wide range of citrus flavoured soft drinks and candy products [59]. We performed an SV-AUC analysis of the BSM/*p*-cresol mixture (Fig. 5a) and it was revealed that the sedimentation coefficient distribution of BSM showed a small degree of change to lower s values. Interestingly an additional Raman analysis revealed a decrease in intensity of the amide I region (~1640 cm^−1^) upon addition of *p-*cresol, along with an increase in the ~850 cm^−1^ region, corresponding to the free *p*-cresol present in the solution [60] (Fig. 5b).

## Concluding Remarks

Our study shows that bovine submaxillary mucin has a broad molecular weight distribution with weight average (700 + 30) kDa and adopts a randomly coiled conformation at neutral pH 7.0 – consistent with mucins from other sources [39] which is maintained under high acid conditions at pH 3.0. However there is a clear increase in aggregation at the lower pH causing a significant shift in the weight average molecular weight to (1600 + 160) kDa. There is also a small change in the sedimentation coefficient distribution on addition of p-creosol with a concomitant small change in the Raman spectrum: this observation may also have affect in flavour release.

These effects have not been investigated previously, and it could lay grounds for new research into the health impact of commercially available flavour compounds. While the compounds used in our study are found in food or drink, they are present at very low concentrations (parts per million/billion) and are not high enough to elicit a significant effect. However, these mechanisms may have significant health implications in other products such as flavoured electronic cigarettes, where their aroma concentrations of be up to a million times higher.
